# Management of severe COVID-19 patient with negative RT-PCR for SARS-CoV-2: Role of clinical, radiological, and serological diagnosis

**DOI:** 10.1016/j.radcr.2021.03.049

**Published:** 2021-03-30

**Authors:** Soedarsono Soedarsono, Anna Febriani, Helmia Hasan, Anita Widyoningroem

**Affiliations:** a Department of Pulmonology and Respiratory Medicine, Faculty of Medicine, Universitas Airlangga, Surabaya, Indonesia; b Department of Radiology, Faculty of Medicine, Universitas Airlangga, Surabaya, Indonesia; c Dr. Soetomo General Hospital, Surabaya, East Java, Indonesia

**Keywords:** Coronavirus Disease 2019, Severe Acute Respiratory Syndrome Coronavirus 2, Real-Time RT-PCR, False-Negative

## Abstract

A real-time reverse transcriptase polymerase chain reaction (RT-PCR) is the gold standard in diagnosis for infection of severe acute respiratory syndrome coronavirus 2 (SARS-CoV-2), but the false-negative result is the problem in the prevention and control the pandemic of coronavirus disease 2019 (COVID-19). A false-negative of RT-PCR test needs to be evaluated when the patient showed a high clinical suspicion for COVID-19. We report a 36-year-old man with 4 times negative RT-PCR results, but clinical, radiological (chest X-ray and chest CT scan), and serological examinations showed a high suspicion of COVID-19. History of close contacted with COVID-19 confirmed patient was reported, and the wife of our case was also RT-PCR tested positive for SARS-CoV-2 in the next few days strengthen the COVID-19 diagnosis of our case patient. It is important to use the combination of RT-PCR, chest X-ray, chest CT scan, clinical manifestations, antibodies test, and exposure history of patients to diagnose COVID-19 and decide the early isolation and appropriate treatment.

## Introduction

Coronavirus disease 2019 (COVID-19) caused by severe acute respiratory syndrome coronavirus 2 (SARS-CoV-2) has been declared as a global pandemic by the World Health Organization (WHO) [1]. COVID-19 is a viral disease that can affect every age group, resulting in a wide spectrum of various clinical manifestations [2]. Currently, reverse transcriptase polymerase chain reaction (RT-PCR) based diagnostic tests (which detect viral nucleic acids) are considered the gold standard for detecting current SARS-CoV-2 infection [3,4]. SARS-CoV-2 infection poses several diagnostic challenges. However, many studies reported the lower sensitivity of the current recommendations on COVID-19 diagnosis and the accurate diagnosis is still being questioned [5]. Patients with a high clinical suspicion for COVID-19 can sometimes have multiple negative tests [6]. The false-negative PCR result has been pushed significant attention and confused the clinicians as final diagnosis relies on RT-PCR positivity for the presence of SARS-CoV-2 in sufficient quantify [7,8,9].

False-negative RT-PCR results can have consequences of failure to quarantine the infected patient, increased the risk of transmission, and possible to cause mortality [9]. False-negative RT-PCR results could hamper the prevention and control of the pandemic, especially because this test is the reference in deciding to continue the medical-observed isolation or discharge [10]. The combination of additional methods for COVID-19 diagnosis is urgently needed in this stage of pandemic. We herein report a 36-year-old man patient who had a history of exposure to COVID-19 confirmed patient. He was admitted to our hospital with clinical, radiological, and serological findings of COVID-19 but RT-PCR test repeatedly showed negative results, while his wife tested positive for SARS-CoV-2 using RT-PCR in the next following days.

## Case presentation

**Fig. 1:**
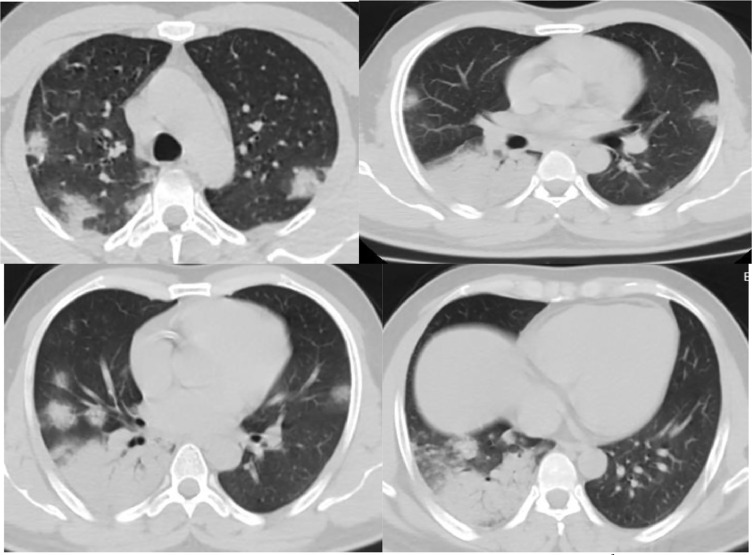
Chest CT scan on 1st day of hospitalization, 3rd day of illness.

**Fig. 2:**
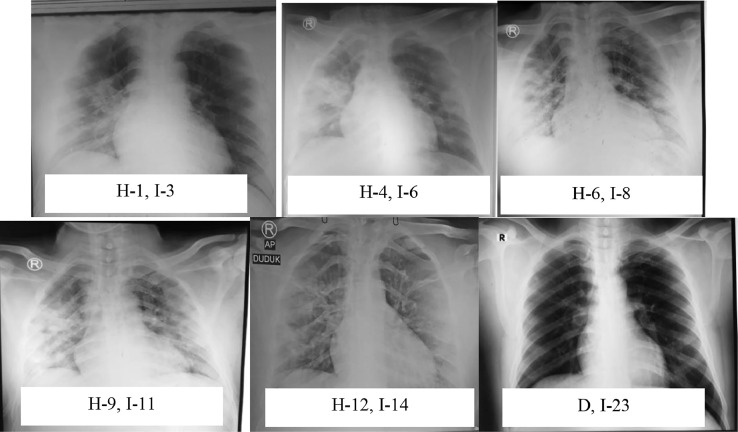
Serial of chest X-ray and chest CT scan. H = day of hospitalization, I = day of illness, D = discharged from isolation room.

A 36-year-old man was hospitalized due to persistent fever and fatigue from the last 2 days, dry cough from the last 3 days, and nausea. Sore throat and dyspnea were not presented. He reported history of close contact with COVID-19 confirmed patient. This patient has co-morbidity of heart disease with old myocardial infarct with ring stent placement. The physical examination revealed a body temperature of 37.8°C, blood pressure of 130/85 mm Hg, pulse rate of 108 beats per minute, and respiratory rate of 22 breaths per minute. SpO_2_ was 97% with simple mask oxygenation 6 lpm (Table 1). Clinical laboratory examination on hospital day-1 showed a normal lymphocyte 23.9% and procalcitonin (PCT) 0.04 ng/mL (Table 1). On 1st day of hospitalization, first examination of chest X-ray showed multifocal rounded opacities in the right lung and chest CT scan showed patchy peripheral consolidation with ground glass opacity (GGO) in both lung and extensive consolidation with air bronchogram on inferior lobe of the right lung suggestive of viral pneumonia (Fig. 1 and 2).

**Table 1:** Summary of clinical features, laboratory, RT-PCR, and serological results.

**Day of illness**		**1**	**2**	**3**	**4**	**5**	**7**	**9**	**10**	**13**	**14**	**24**
**Day of hosp**				**1**	**2**	**3**	**5**	**7**	**8**	**11**	**12**	**21**

Fever			Y	Y	Y							
Temperature (°C)				37.8	37.8							
Dry cough		Y	Y	Y	Y							
Myalgia					Y							
Nausea					Y							
Vomite									Y	Y		
SpO_2_				97% (SM)								
Nasopharyngeal swab (RT-PCR)				Neg	Neg		Neg				Neg	
IgM Anti SARS CoV-2				NR				R				R
IgG Anti SARS CoV-2				NR				R				R

	Reference Range											

Hb (g/dL)	13.3-16.6			15.3					14.1	13.9		
WBC (10^3/uL)	3.37-10.0			4770					6620	13190		
Limfosit (%)	23.1-49.9			23.9					21.6	14		
CRP (mg/dL)	0-1			1.7						1.5		
D dimer (ng/mL)	<500					1550			1060	840		
Ferritin (ng/mL)	22-322				2356					1355		
PCT (ng/ml)	<0.5			0.04			0.09			0.08		

Y=yes, Neg=negative, NR=nonreactive, R=reactive, SM=Simple Mask Oxygenation.

A diagnosis of suspect COVID-19 pneumonia was made. However, the samples from nasopharyngeal and oropharyngeal swabs which were tested using *Real-Time Polymerase Chain Reaction* (RT-PCR) for SARS-CoV-2 showed negative (Table 1). Considering the diagnosis of suspect COVID-19 pneumonia, this patient was admitted in the isolation room and treated with O_2_ supplementary 6 L/min by simple mask, high in calories and high in protein dietary 2100 kcal/24 hours, NaCl infusion 0.9% 1500 mL/24 hours, multivitamins containing vitamin C, Vitamin D, Zinc, N-acetylcystein 600 mg every 12 hours po, and paracetamol 500 mg po every 6 hours. Therapy for co-morbidity is continued with bisoprolol 2.5 mg every 24 hours, acetyl salicylic acid (ASA) 100 mg every 24 hours, and atorvastatin 20 mg every 24 hours.

**Fig. 3:**
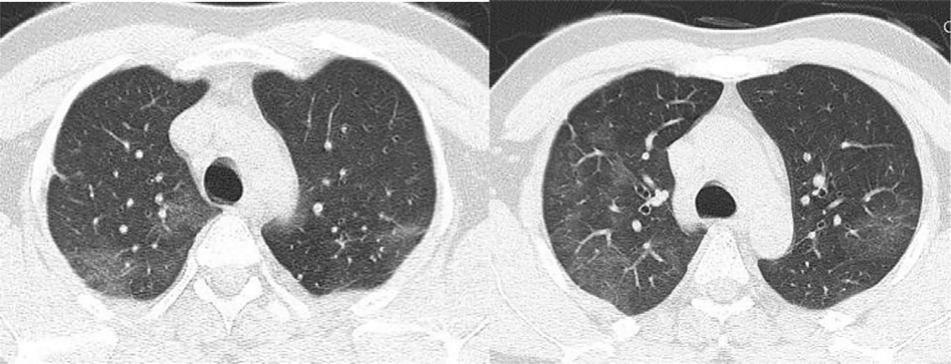
Chest CT scan on 30th day of illness.

*Real-Time Polymerase Chain Reaction* for SARS-CoV-2 was repeated on the 2nd, 5th, and 12th day of hospitalization and remain showed negative (Table 1). This was contradictory to his symptoms and radiographic findings, which were characteristics for viral pneumonia. Chest X-ray on 4th day of hospitalization showed addition of infiltrates in the right and new infiltrate in the left lung (Fig. 2). The following therapy were oseltamivir 2 × 75 mg for 7 days, methisoprinol 3 × 500 mg po for 5 days, dexamethasone injection 1 × 6 mg IV for 6 days, enoxaparin sodium 1 × 0.6 mL subcutan for 8 days. While our case patient is admitted in the isolation room with RT-PCR negative for SARS-CoV-2 and radiographic showed viral pneumonia, his wife reported RT-PCR positive for SARS-CoV-2 with no symptoms (asymptomatic). Our case patient was tested for serological antibodies on the 7th day of hospitalization to confirm the suspected diagnosis. Both IgM and IgG for SARS-CoV-2 showed reactive, serological positive results. This result made clear that this patient had COVID-19 infection. Serial chest X-ray from 1st to 12th day of hospitalization showed a worsened appearance of lungs, but then it improved progressively and eventually went back to normal healthy lungs (Fig. 2) and also clinical symptoms were resolved. This patient discharged from isolation room on next day and admitted in the nonisolation room. Antibodies test was repeated after discharged from isolation room. Both IgM and IgG for SARS-CoV-2 remain showed reactive (Table 1), while chest X-ray showed no more infiltrate (Fig. 2). Evaluation of chest CT scan on 30th day of illness showed multi focal GGO in peripheral both lung (reabsorption stage of COVID-19 pneumonia) (Fig. 3). The patient continued self-isolation for 14 days. He returned to his daily activities after completed self-isolation under the clinician consideration and based on the results of clinical and radiological.

## Discussion

Fever and dry cough are reported as the most common respiratory symptoms in COVID-19 patients [11]. Fatigue (11-44%) and nausea (7%) are also common reported as nonrespiratory symptoms of COVID-19 [2,11,12]. A high spread of SARS-CoV-2 results various clinical manifestations, even different clinical manifestations in a family cluster of COVID-19 [13]. Clinician decided to diagnose as suspected COVID-19 and started to isolate and treat him as COVID-19 patient due to a high suspicion of COVID-19 according the clinical, radiological, and serological examinations, although 4 times RT-PCR from nasopharyngeal swabs showed negative for SARS-CoV-2.

Chest CT scan which showed patchy peripheral consolidation with ground glass opacity (GGO) in both lung and extensive consolidation with air bronchogram on inferior lobe of the right lung (Fig. 1), and bilateral multi focal patchy opacity in serial examination which deterioration especially on the 4th CXR (Fig. 2) suggested the presence of viral pneumonia (Fig. 1). According to NIH guideline (2020), abnormalities seen in CT typically reveal bilateral peripheral ground-glass opacities with the development of areas of consolidation later in the clinical course [3]. The most common patterns on chest CT were ground-glass opacity (56.4%) and bilateral patchy shadowing (51.8%) [14]. Chest X-ray is one of the important, noninvasive clinical adjuncts that play an essential role in the preliminary investigation of different pulmonary abnormalities. It can act as an alternative screening modality for the detection of COVID-19 or to validate the related diagnosis [15].

The role of chest CT scan in the identification and management of COVID-19 patients with false-negative RT-PCR results has been highlighted [5]. In a cohort of patients with COVID-19 infection and imaging follow-up, baseline chest radiography had a sensitivity of 69% compared with 91% for initial RT-PCR testing. Abnormalities at chest radiography were able to be depicted in 6 patients whose initial RT-PCR was negative for COVID-19 (6 of 64; 9%) [16]. A ground-glass opacification was noted in most patients from the very beginning of symptoms. At presentation, CT sensitivity was therefore 97.2%, whereas the sensitivity of initial rRT-PCR was only 83.3%. Patients with typical CT findings but negative rRT-PCR results should be isolated and rRT-PCR should be repeated [8].

*Real-Time Polymerase Chain Reaction* is the gold standard for detecting SARS-CoV-2 infection [3,4], which relies on sufficient viral levels for gene amplification [6]. SARS-CoV-2 infection poses several diagnostic challenges, especially when negative RT-PCR was obtained but clinical and radiographic findings showed a high suspicion and consistent with COVID-19. False-negative RT-PCR results have been attracted significant attention recently in many reports [6,7,10,17]. The sensitivity of the RT-PCR assay is dependent on viral load with peak levels occurring on day 4 based on virological studies. The reliability of the test is limited by the timing of sample collection with regards to symptom onset and user technique with nasopharyngeal swabs [6]. RT-PCR method heavily relies on the presence of the viral genome in sufficient amounts at the site of sample collection that can be amplified. Missing the time window of viral replication can provide false-negative results. An incorrect sample collection also can limit the usefulness of the PCR assay [9]. According to the time of the symptom onset of our case, the course of illness should have enough to test positive. The negative result of our case was possible caused by clinician sampling error or the low positive rate of the specimen. Li et al. (2020) reported a low sensitivity of RT-PCR tests with only 39.5% cases had at least one positive RT-PCR result from total 610 patients COVID-19 confirmed diagnosis using the combination of clinical and radiological findings [10].

Lower respiratory tract specimens are considered to have higher yield, due to high viral load, and should be obtained whenever possible if there is diagnostic uncertainty regarding COVID-19 [3]. Positive rate of BALF was 93.3%, followed by sputum (72.1%), while nasal swabs was 62.5%, and pharyngeal swabs was 31.7% [18]. In our case, the clinician decided to not use samples from BAL and sputum induction due to a high risk of aerosol generation [3]. Additional diagnosis methods can be highly beneficial to ensure timely diagnosis of the infected patient with a false-negative RT-PCR [9]. COVID-19 treatment in this case was continued. Serological test of IgM and IgG showed reactive on 9th day of illness also supported our decision. A study reported the median duration of IgM antibody detection was 5 days, while IgG was detected 14 days after symptom onset, with a positive rate of 85.4% and 77.9%, respectively. The positive detection rate is significantly increased (98.6%) when combining IgM ELISA assay with PCR for each patient compared with a single quantitative PCR test (51.9%) [9].

A study reported that there was no statistically significant difference in the recovery time for RT-PCR and chest radiography recovery groups (*P* = .33) in their mean durations to recovery [16]. Our case patient showed an improvement of the clinical symptoms and serial chest X-ray from 1st to 12th day of hospitalization (Fig. 2). He was discharged from isolation room and admitted in the nonisolation room. However, the clinical, radiological, and serological tests of this case which showed high suspicion of viral pneumonia does not rule out the possibility of viral pneumonia other than COVID-19, but considering the ongoing COVID-19 pandemic, we decided for early isolation and COVID-19 treatment for this patient. Decided for diagnosis of COVID-19 was also supported with the RT-PCR result of the wife of this case which showed positive for SARS-CoV-2 and possibly transmitted by our case patient.

## Conclusion

The clinician should be aware with false-negative RT-PCR results, although nasopharyngeal RT-PCR is a gold standard for COVID-19 diagnosis. The diagnosis of COVID-19 should be made using the combination of RT-PCR, chest X-ray, chest CT scan, clinical manifestations, antibodies test, and exposure history of patients. This consideration is urgently required to identify, isolate, and treat the patients as soon as possible to reduce mortality rates and the risk of transmission.

## Ethics approval and consent to participate

We are exempt from ethical approval from Dr. Soetomo Hospital Institutional Review Board as it is not required in our hospital for a single case report.
